# *Saccharomyces cerevisiae* I4 Showed Alleviating Effects on Dextran Sulfate Sodium-Induced Colitis of Balb/c Mice

**DOI:** 10.3390/foods11101436

**Published:** 2022-05-16

**Authors:** Yuan Meng, Lijuan Zhang, Panpan Li, Jiang Yu, Guangqing Mu, Xinling Li, Yanfeng Tuo

**Affiliations:** 1 School of Food Science and Technology, Dalian Polytechnic University, Dalian 116034, China; my_2311675814@163.com (Y.M.); 15386843201@163.com (L.Z.); lpp1293318974@163.com (P.L.); yujiang0324@163.com (J.Y.); gq6552002@aliyun.com (G.M.); 2 Dalian Probiotics Function Research Key Laboratory, Dalian Polytechnic University, Dalian 116034, China; 3 Xinjiang Tianrun Biological Technology Co., Ltd., Urumchi 830011, China; xjlxl911@163.com

**Keywords:** *Saccharomyces cerevisiae* I4, probiotic, intestinal flora, intestinal barrier, SCFAS

## Abstract

Ulcerative colitis (UC) is a chronic inflammatory bowel disease. The purpose of this study was to investigate the ameliorating effects of three yeast strains, *Saccharomyces cerevisiae* I4, *Clavispora lusitaniae* 30 and *Pichia kudriavzevii* 11, isolated from traditional fermented dairy food in Xinjiang, China, on the ulcerative colitis symptoms of Balb/c mice treated by dextran sulfate sodium (DSS). Among which, *S. cerevisiae* I4 had good tolerance to simulated gastrointestinal juice and strong adhesion to HT–29 cells monolayers. Furthermore, the three yeast strains were oral administered to Balb/c mice with DSS induced colitis. The weight loss, colon shortening and histological injury of colitis mice were ameliorated. Then, oral administration of *S. cerevisiae* I4 improved the immune state by reducing the contents of TNF–α, IL–6 and IL–1β and increasing immunoglobulin. The relative expression of intestinal barrier proteins Claudin–1, Occludin and Zonula Occludins–1 (ZO–1) of the mice enhanced, and the short chain fatty acids (SCFAs) content such as Propionic acid, Butyric acid, Isobutyric acid and Isovaleric acid in the feces of the mice increased to varying degrees, after *S. cerevisiae* I4 treatment compared with the model group of drinking 3% DSS water without yeast treatment. Moreover, *S. cerevisiae* I4 treatment lifted the proportion of beneficial bacteria such as Muribaculaceae, Lactobacillaceae and Rikenellaceae in the intestinal tract of the mice, the abundance of harmful bacteria such as *Staphylococcus aureus* and *Turicibacter* was decreased. These results suggested that *S. cerevisiae* I4 could alleviate DSS induced colitis in mice by enhancing intestinal barrier function and regulating intestinal flora balance.

## 1. Introduction

Ulcerative colitis (UC) is a type of inflammatory bowel disease (IBD), which has a high incidence and prevalence worldwide and is characterized by recurrent and uncontrollable inflammation [1]. IBD also includes another kind of intestinal disease called Crohn. Their attacks can cause a variety of gastrointestinal injuries, usually manifested as recurrent and persistent abdominal pain, diarrhea, bloody stool and other symptoms [2,3]. In the early stage of the disease, the integrity of the intestinal epithelium is destroyed, various harmful bacteria and their metabolites penetrate the intestinal barrier, leading to abnormal immune responses and inflammation [4]. The pathogenesis of colitis is a hotspot of current research. Although it is not clear yet, there is evidence that the onset of colitis is related to the destruction of intestinal epithelial barrier and the disorder of intestinal gut microbiota [5,6].

The intestinal epithelial barrier is the first line of defense against the invasion of various foreign pathogens or toxins. It is chiefly composed of intestinal epithelial cells, mucous membrane and tight junction proteins [7]. Tight junction proteins (TJs), which mainly includes a variety of functional proteins, such as Claudin–1, Occludin and ZO–1 [8], are the key to maintain the permeability between intestinal epithelial cells. Once the intestinal barrier is breached, various inflammatory bowel diseases are accelerated by increased intestinal permeability. In addition, there are a wide variety of bacteria in the intestinal tract of both human and other organisms. The interaction between them and the host forms an intestinal microecosystem that can exchange information and materials in the long-term evolution process [9]. The gut microbiota in a healthy intestinal maintains a dynamic balance. Abnormal changes in the types and quantities of normal intestinal flora will lead to the disorder of gut microbiota, and the occurrence of colitis is closely related to the gut microbes dysbiosis [10,11].

A large amount of evidence showed that probiotics have a relieving effect on ulcerative colitis as biological agents [12]. Disruption of intestinal gut microbiota and damage to the integrity of the intestinal barrier are major causes of colitis, and probiotics intake has been shown to be effective in dextran sulfate sodium-induced colitis mice [13,14,15]. Shuang Yan et al. demonstrated that an extracellular polysaccharide producing *Bifidobacterium longum* YS108R alleviates DSS–induced colitis of mice evidenced by the increased expression of tight junction proteins and mucins in colon tissue and reduced abundance of Proteobacteria in intestinal gut microbiota [16].

*Lactobacillus* probiotics are normally the most common choice for the treatment of colitis, while few studies have focused on yeast which is subordinate to fungi. The inhibition against harmful bacteria, immune regulation and inflammation relief functions of yeast strains from different sources has been studied. Some yeast strains exhibited certain beneficial functions, and even have more advantages than lactic acid bacteria in some aspects [17,18]. Probiotic properties such as producing a variety of enzymes, being more resistant than lactic acid bacteria, containing a unique β–glucan component in its cell wall, and being used in the treatment of antibiotic-associated diarrhea make it also meet the main criteria and requirements for the definition of probiotics [19]. It has been reported that *Saccharomyces boulardii* preparation has a good prevention and treatment effect on traveler’s diarrhea, intestinal disorders caused by antibiotics and other diseases, which has great potential in the treatment of inflammatory bowel disease [20,21,22]. Studies have shown that daily use of a certain amount of *Saccharomyces boulardii* for six months can significantly reduce the recurrence of Crohn’s disease [23].

In recent years, *S. boulardii* has attracted more attention in clinical application, but whether other kinds of yeasts have the function of relieving colitis remains to be proved. In this study, Balb/c mice with colitis induced by DSS were used as a model to explore the effect and mechanism of three different kinds of yeast strains, *Saccharomyces cerevisiae* I4, *Clavispora lusitaniae* 30 and *Pichia kudriavzevii* 11, on colitis, aiming to provide new ideas for further exploring the probiotic properties of yeast. *S. cerevisiae* I4, *C. lusitaniae* 30 and *P. kudriavzevii* 11 were isolated from traditional fermented dairy food in Xinjiang, China. In addition to lactic acid bacteria, there are some species of yeast in the traditional fermented dairy food from Xinjiang, which has a long history of consumption by local residents.

## 2. Materials and Methods

### 2.1. Culture Conditions of Yeast Strains

The three yeast strains, *Saccharomyces cerevisiae* I4, *Clavispora lusitaniae* 30 and *Pichia kudriavzevii* 11, used in this study were isolated from traditional fermented dairy food and stored in Dalian Probiotics Function Research Key Laboratory, Dalian Polytechnic University. All the yeast strains were cultured in liquid YPD medium at 37 °C for 24 h. After three generations of activation, the cell pellets were obtained by centrifugation (4 °C, 3500 r/min, 10 min), and then the new YPD medium was added for re–suspension for subsequent experiments.

### 2.2. Survival Rate of Yeasts in Simulated Gastric and Intestinal Juice

To test the survival rates of each yeast under gastric and intestinal conditions, three yeast strains were exposed to simulated artificial gastric solution with pH 2.5 and 0.3% pepsin (*w*/*v*) for 3 h and intestinal juice with pH 8.0 and 1.8% bovine bile salt and 0.1% trypsin (*w*/*v*) for 8 h [24]. The survival rate (CFU/mL) of yeast strains in gastrointestinal fluid was counted by pouring plate method through YPD solid medium, which was calculated as follows:

(1) $$\mathrm{Survival}\mathrm{rate}\left(\%\right)=\frac{\mathrm{Log}N_{t}}{\mathrm{Log}N_{0}}\times 100$$

where *N_t_* represents the number of viable count (CFU/mL) treated with simulated gastric or intestinal fluids and *N*_0_ represents the initial viable count of the strains. 

### 2.3. Adhesion to HT–29 Cell Monolayers

Human colonic cancer cell line HT–29 was obtained from Chinese Academy of Science (Shanghai, China). HT–29 cells were cultured in RPMI 1640 medium supplemented with 10% (*v*/*v*) fetal calf serum (inactivated at 56 °C for 30 min, Sijiqing Co. Ltd., Hangzhou, China) and 1% penicillin–streptomycin (Gibco, Waltham, MA, USA) at 37 °C with 5% CO_2_ incubator. When the coverage area of HT–29 cells reached 80–90% of the culture flask, the cells were digested with trypsin (Gibco, Burlington, ON, Canada).

HT–29 cells above digested with trypsin were inoculated with 2.5 *×* 10^5^ cells/mL in 24 well plates when the cells grew to a certain number and incubated for 48 h at 37 °C for the subsequent adhesion experiment as previously described and modified as needed [25]. *S. cerevisiae* I4, *C. lusitaniae* 30 and *P. kudriavzevii* 11 were re-suspended in RPMI–1640 medium without fetal bovine serum and antibiotics and prepared at a concentration of 1 *×* 10^7^ CFU/mL, respectively. The 24 well–plate was washed 3 times with phosphate buffer saline (PBS) before the yeast solution was added. After treated with the three yeast strains for 2 h, HT–29 cells were washed 3 times with PBS to remove unbound yeasts and lysed with 0.5% Triton X-100 (Sigma) completely. Then adhering yeasts were plated on YPD agar medium to determine the number of colony-forming units (CFU) after appropriate dilutions in physiological saline.

(2) $$\mathrm{Adhesion}\mathrm{rate}\left(\%\right)=\frac{\mathrm{Log}N_{1}}{\mathrm{Log}N_{0}}\times 100$$

where: *N*_1_ represents the colony number of yeast adhering to HT–29 cells; *N*_0_ represents the colony number of yeast in the initial suspension;

### 2.4. Cytotoxity Assay

The method described by Felice et al. was slightly modified to perform the cytotoxicity assay [26]. HT–29 cells were seeded at 2.5 *×* 10^5^ cells per well in 96–well plates and cultured at 37 °C in a 5% CO_2_ cell incubator for 24 h. After washing twice with PBS, the yeast pellets were re–suspended with RPMI 1640 blank medium without serum and antibiotics, and the concentration was adjusted to 1 *×* 10^7^, 10^6^ and 10^5^ CFU/mL per well before put into the plates, while the control group was just added with 100 µL above blank medium. After 24-h cultivation, PBS was used to wash away the residue, and 50 µL methylene blue staining solution (98% HBSS, 0.67% glutaraldehyde and 0.6% methylene blue) was added to each well. After incubation at 37 °C for 1 h, culture plates were washed 6–7 times with deionized water and blow–dried in a fume hood, then 100 µL of decolorizing solution (1% glacial acetic acid, 49% 1 *×* PBS and 50% anhydrous ethanol) was added to each well and decolorized for 20 min on a shaker. The blank well without cells was added with 100 µL of decolorizing solution and the absorbance of each well at 570 nm was finally measured.

### 2.5. Effect of Yeast on Tight Junction Protein Expression in HT-29 Cell Monolayers

Determination of tight junction proteins was referred to the method of Gao et al. [27]. HT–29 cells were inoculated with 4 *×* 10^5^ cells/well in 6–well plates for 48 h. Three yeast species (re–suspended with RPMI–1640 medium without fetal bovine serum and antibiotics), including *S. cerevisiae* I4, *C. lusitaniae* 30 and *P. kudriavzevii* 11 adjusted to 1 *×* 10^7^ CFU/mL were added and incubated for 2 h, then the supernatant was discarded and washed twice with PBS. Under the condition of ice bath, the bottom protein was scraped by a cell scraper and its concentration was determined by bicinchoninic acid (BCA) kit after pretreatment. After separated by SDS gel electrophoresis, the protein was transferred to polyvinylidene fluoride (PVDF) membrane by wet method. Then the membranes were incubated with Occludin, Claudin–1, ZO–1 and β–actin antibody (diluted as 1:1500) at 4 °C overnight and further incubated with corresponding secondary antibody at a dilution of 1:2000. At last the protein bands were visualized by Meilunbio ECL Luminescent Solution. β-actin was used to be the internal control protein.

### 2.6. Establishment of Animal Model of Colitis

6–8 weeks old SPF Balb/c female mice were purchased from Liaoning Changsheng Biotechnology Company (Benxi, China) and adaptively fed for one week (conditions: humidity in the room is 50 ± 10%, temperature is 22 ± 1 °C, 12 h day and night alternately). Meanwhile, they were allowed to drink and eat freely. After one week of adaptive feeding, 50 mice were randomly divided into 5 groups with 10 mice in each group. It includes control group (CT), DSS model group (DSS), *C. lusitaniae* 30 group (CL), *P. kudriavzevii* 11 group (PK) and *S. cerevisiae* I4 (SC) group. All the mice were given pure drinking water freely in the former seven days. The mice in blank group and model group were given 0.9% sterile saline by gavage at the same time, while the remaining three groups were administered with 0.2 mL corresponding yeast solution with a concentration of 1 *×* 10^7^ CFU/mL. The pure drinking water was replaced with the water containing 3% DSS for the mice in DSS model group, *C. lusitaniae* 30 group, *P. kudriavzevii* 11 group and *S. cerevisiae* I4 group during the later seven days to induce colitis, except the mice in the blank group. In addition, the drinking water of mice in each group was changed every 2 days. All animal experiments were carried out in accordance with the Guidelines of the Experimental Animal Ethics Committee of Dalian Polytechnic University (SYXK2017–0005).

### 2.7. Assessment of Disease Activity

Bodyweight change, stool consistency and occult blood in stool were recorded every day as previously reported [28]. Weight loss was scored as follows: no weight loss = 0, weight loss from baseline 1–5% = 1, 5–10% = 2, 10–20% = 3, more than 20% = 4. For stool viscosity, 0 point for well–formed particles, 2 points for paste and semi formed feces, and 4 points for water feces. For bloody stool, 0 point for no blood, 2 points for a little bleeding and 4 points for massive bleeding. These scores were added and divided by 3 to obtain DAIs ranging from 0 (healthy) to 4 (most severe colitis).

### 2.8. Histopathological Analysis

The distal colon was taken and washed with sterile normal saline solution for histological analysis. About 1 cm colon tissue was cut and fixed in 10 times diluted formaldehyde solution, then it was dehydrated with graded alcohol solution (75–100%), embedded in paraffin and sliced for hematoxylin eosin staining and alcian blue staining, the rest of the colon tissue was preserved according to other experimental requirements.

### 2.9. Determination of Immune Related Factors and Inflammatory Mediators

Mice were sacrificed after 12 h of water and food deprivation and weighed before being euthanized, the liver, spleen and thymus were removed and weighed to calculate the organ index. The calculation formula is as follows:

(3) Organindex=organweight(g)/bodyweight(g)

Blood was obtained from the orbit of mice and the supernatant was collected by centrifugation at 4 °C. The relative contents of inflammatory factors IL–8, IL–10, IL–1β and TNF–α and immunoglobulin IgM, IgA and IgG in serum were determined by the corresponding kit according to the instructions.

### 2.10. Genomic DNA Extraction and PCR Amplification

The genomic DNA of the fecal samples of the mice in each group was extracted by CTAB method, and the purity and concentration of DNA were detected by agarose gel electrophoresis [29]. Primers of 16S V3-V4 region (341F CCTAYGGGRBGCASCAG and 806R GGACTACNNGGGTATCTAAT) were selected for PCR by Phusion^®^ High-Fidelity PCR Master Mix with GC Buffer (New England Biolabs, Ipswich, MA, USA). The PCR procedure was as follows: Initial denaturation at 94 °C for 5 min, denaturation was performed 30 times at 95 °C for 30 s, annealing at 55 °C for 50 s and extension at 72 °C for 30 s, then the final extension was at 72 °C for 5 min. PCR products were detected by 2% agarose gel electrophoresis. The library was constructed using TruSeq^®^ DNA PCR-Free Sample Preparation Kit and was quantified by Qubit and Q-PCR, then sequenced using NovaSeq6000 (Beijing Novogene Bioinformation Science and Technology Co., Ltd., Beijing, China). The reads of each sample were spliced and filtered using FLASH (V1.2.7, https://ccb.jhu.edu/software/FLASH/, accessed on 7 September 2011) according to Barcode sequence and PCR amplification primer sequence. All Effective Tags of the samples are clustered using Uparse algorithm (Uparse v7.0.1001, http://www.drive5.com/uparse/pairs), and the sequence is clustered as OTUs by default with 97% identity. Mothur method and SILVA132 (http://www.arb-silva.de/) SSUrRNA database were used to annotate OTUs sequences. Chao1, Shannon, Simpson, and ACE indices were calculated using Qiime software (Version 1.9.1, Knight and Caporaso labs, Northern Arizona University, Flagstaff, AZ, USA) to assess sample diversity and richness. PCoA, NMDS (R software, Version 2.15.3) and linear discriminant analysis (LEfSe Software) were used to compare the differences in microbial community composition between groups of samples.

### 2.11. Western Blotting

Western Blot analysis was carried out as previously described [12]. The colon tissue proteins of the mice in each group were homogenized with RIPA lysis buffer (Beijing Solarbio Science and Technology Co., Ltd., Beijing, China) for protein extraction. The extracted proteins were subjected to SDS-PAGE electrophoresis (concentrated gel current of 8 mA/gel and separated gel current of 16 mA/gel) with 20 μL sample per well. After that, the proteins were transferred to PVDF membrane and incubated with primary antibody (diluted as 1:1500) including Claudin–1, Occludin, ZO–1 and β–actin against at 4 °C overnight. The secondary antibody (diluted as 1:2000) labeled with horseradish peroxidase was incubated for 1 h the next day. After TBST washing, the protein bands were visualized by Meilunbio hypersensitivity ECL luminescence solution and quantified by Image J software with β-actin as the internal reference protein.

### 2.12. Analysis of Short Chain Fatty Acids (SCFAs) Content

The relative concentrations of SCFAs including Acetic acid, Propionic acid, Isobutyric acid, Butyric acid, Isovaleric acid, Valeric acid, Caproic acid in the fecal samples of the mice in each group were measured by gas chromatography mass spectrometry (GC-MS, Beijing NovogeneCo., Ltd., Beijing, China) [30]. Ether was used to prepare seven short-chain fatty acids into standard solutions with different concentrations to make standard curves. 50 μL 15% phosphoric acid mixed 100 μL of 125 μg/mL internal standard (Isohexic acid) solution and 400 μL ether were added to a 2 mL centrifuge tube and homogenated for 1 min, the supernatant was obtained after centrifugation (12,000 rpm, 10 min) at 4 °C. Then 1 µL of supernatant through a 0.22 µm filter was injected into a GC (GC2010-plus) equipped with a flame ionization detector and an Agilent HP-INNOWAX column (30 m *×* 0.25 mm, 0.25 µm) by split injection with a split ratio of 10:1.

### 2.13. Statistical Analysis

Statistical analysis was performed using the Statistical Package for Social Sciences (SPSS) software version 20.0.0 (SPSS Inc., Chicago, IL, USA). One–way analysis of variance (ANOVA) with LSD test and Duncan’s multiple range test was used to assess the statistical significance between different groups. All data are expressed as mean ± standard deviation (SD). A value of *p* < 0.05 was considered statistically significant.

## 3. Results

### 3.1. Survival Rate of Yeast Strains in Simulated Gastrointestinal Fluid

Probiotic strains are damaged by the low pH value and pepsin of the stomach, along with bile salt and pancreatin in the small intestine when they go through the gastrointestinal tract, lowering the survival rate of the strains. In order to exert beneficial effect to host, probiotics must have a high viability through the gastrointestinal tract [31,32]. As shown in Table 1, The survival rates of three yeast strains, *S. cerevisiae* I4, *C. lusitaniae* 30 and *P. kudriavzevii* 11 in simulated artificial gastric fluid (pH 2.5) were not significantly different (*p* > 0.05), the survival rates were slightly reduced after being cultured in simulated intestinal fluid (pH 8.0) for 8 h (*p* > 0.05). Under the condition of high concentration of bile salts and enzymes, the survival rates of the three strains reached about 80% in the gastroenteric fluid, indicating that they had a good tolerance to the intestinal environment [33].

### 3.2. The Adhering Ability of Yeast to HT–29 Cells Monolayers

Probiotics need to combine with intestinal epithelial cells after resisting various digestive fluids in order to survive in the host intestinal tract. Adhered strains can enhance their influence on the host health after colonization and further ameliorate the local microbiota or adjust the immune response [34]. As shown in Table 1, the adhesion rates of the three yeast strains on HT–29 cell monolayers reached more than 90%, among which *S. cerevisiae* I4 and *P. kudriavzevii* 11 had relatively stronger adhesion than *C.lusitaniae* 30 (*p* < 0.05).

### 3.3. The Effects of Yeast Strains on the Tight Junction Protein Expression of HT–29 Cell Monolayers

At first, the toxity of different yeast strains under different concentrations to HT–29 cells was determined. As shown in Table 2, the survival rates of HT–29 cells can reach over 90% when exposed to yeast at 10^5^, 10^6^ and 10^7^ CFU/mL, suggesting that the three yeast strains were not toxic to HT–29 cells. Meanwhile, the concentration of 10^7^ CFU/mL was selected for subsequent experiments since the survival rate of HT–29 cells treated with 10^7^ CFU/mL was the highest (*p* < 0.05).

**Figure 1:**
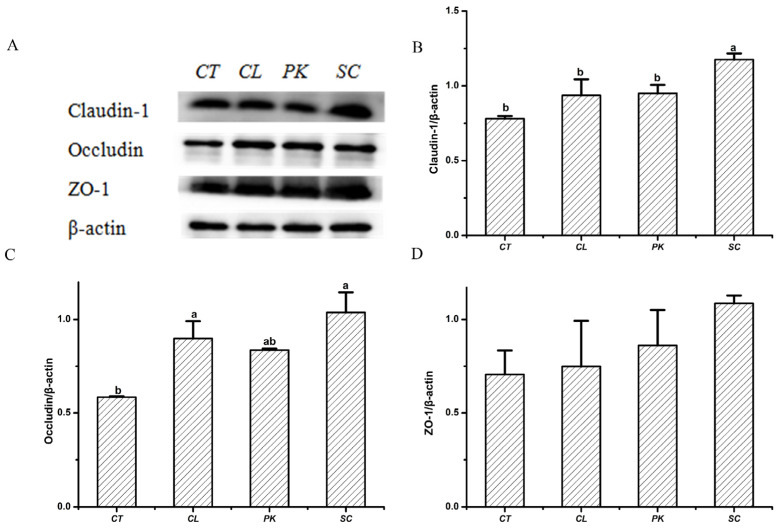
Effects of the yeast strains on the tight junction proteins expression of HT–29 cell monolayers. (**A**) Western blotting images of Claudin–1, Occludin and ZO–1. (**B**) Relative expression of (**B**) Claudin–1, (**C**) Occludin, (**D**) ZO–1. Different lowercase letters marked on the column represent significant difference (*p* < 0.05).

Intestinal tight junction is a permeable intercellular barrier structure, and tight junction proteins are considered to regulate intercellular permeability and play an indispensable role in maintaining intestinal health [35,36]. The expression levels of Claudin–1, Occludin and ZO–1 of HT–29 cell monolayers after exposing to the three yeast strains was measured with β-actin as internal reference. As shown in Figure 1, the relative expression levels of the above proteins augmented after treatment with three yeast strains compared with the blank, among which *S. cerevisiae* I4 had a larger increase (*p* < 0.05), indicating that *S. cerevisiae* I4 and other yeasts could enhance intestinal barrier function by increasing the expression of tight junction proteins.

### 3.4. Alleviating Effect of the Yeast on the Symptoms of Balb/c Mice with Colitis

**Figure 2:**
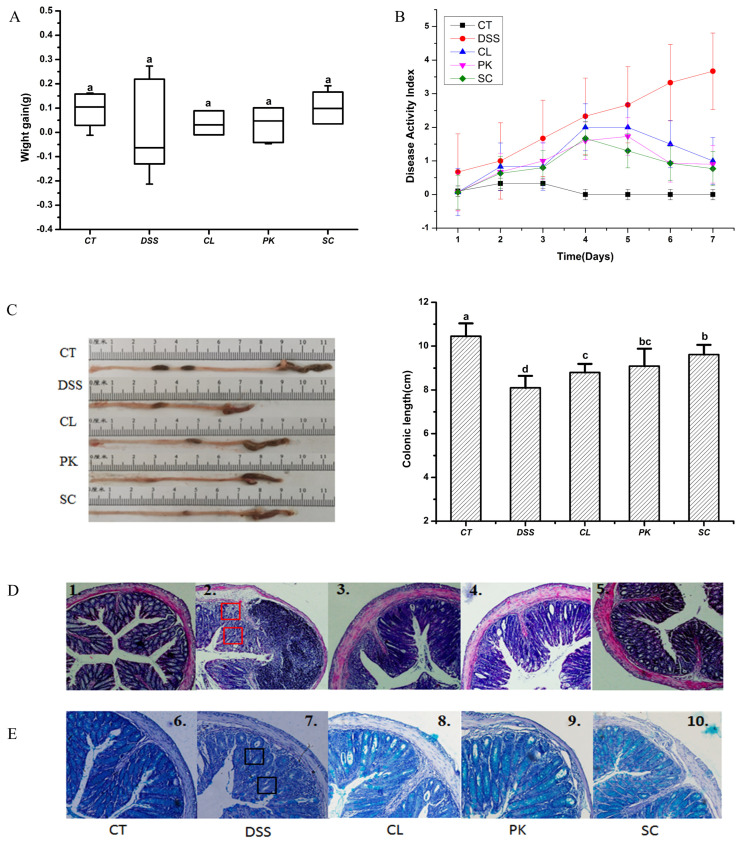
Effects of three yeast strains on symptoms of Balb/c mice with colitis. (**A**) Body weight and (**B**) Disease activity index (DAI) of mice, (**C**) colon morphology and colon length of mice in each group. (**D**) H&E staining (figures 1–5 shows the observation of colonic histological injury of mice in each group). (**E**) Alcian blue staining (figures 6–10 shows the observation of mucin number in colonic tissue of mice in each group). Red marks indicate destruction of crypt structures and inflammatory cell infiltration. Black marks indicate decreased goblet cells and mucins. Different lowercase letters marked on the column represent significant difference (*p* < 0.05).

As shown in Figure 2A, the weight gain of the model group was lower than that of the other groups during the experimental period, although there was no significant difference (*p* < 0.05). While the weight gain of the Balb/c mice in the three yeast groups was close to the blank group, especially in the group oral administration with *S. cerevisiae* I4. In addition, the DAI index of the model group was substantially higher than that of the control group, mainly manifested as loose stool or bloody stool, while the symptoms of the mice treated by DSS were relieved after oral administration of the three yeasts with the decreased DAI score (Figure 2B). DSS induced colitis in mice showed the shortening of colonic length, which is one of the indicators to evaluate the severity of colitis [37,38]. It can be seen from Figure 2C that the colonic length of the Balb/c mice after drinking DSS water was shortened compared with that of the mice in the blank group (*p* < 0.01), while recovered by oral administration of three yeast species (*p* < 0.05). The colon length of the mice in the *S. cerevisiae* I4 group was closest to that in the blank group.

**Figure 3:**
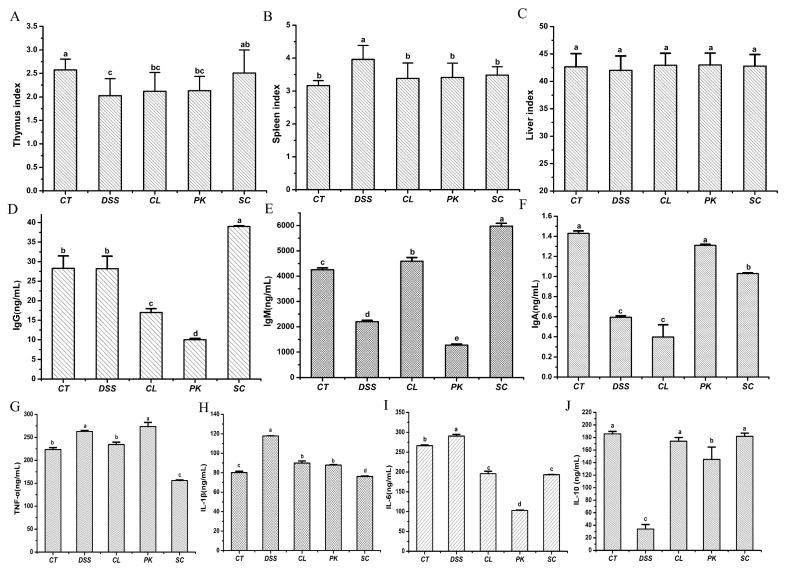
Organ index, contents of immunoglobulin and inflammatory factors in serum of Balb/c mice. (**A**–**C**) Thymus index, Spleen index, Liver index; (**D**–**F**) IgG, IgM, IgA; (**G**–**J**) TNF–α, IL–1β, IL–6, IL–10. Different lowercase letters marked on the column represent significant difference (*p* < 0.05).

**Figure 4:**
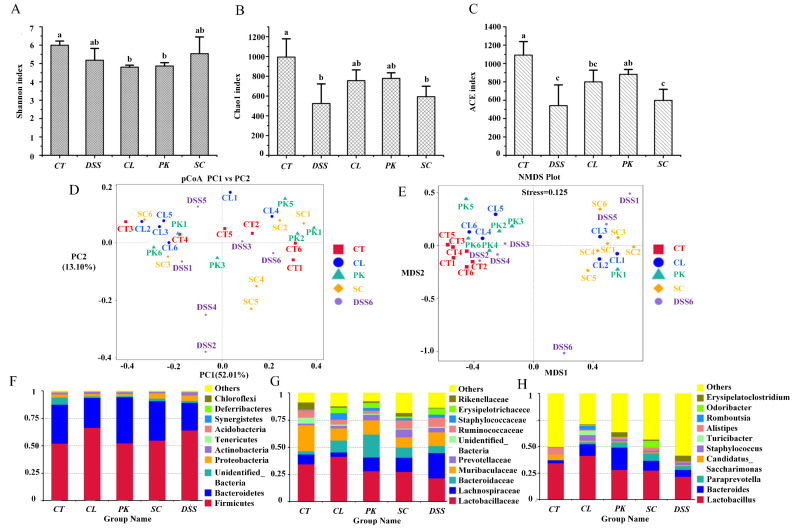
Intestinal microflora diversity and gut microbiota composition of Balb/c mice in different groups. (**A**–**E**) Shannon index, Chao 1 index, ACE index, PCoA, NMDS of each group; bar picture of the gut microbiota at the (**F**) Phylum level, (**G**) Family level, (**H**) Genus level. Different lowercase letters marked on the column represent significant difference (*p* < 0.05).

**Figure 5:**
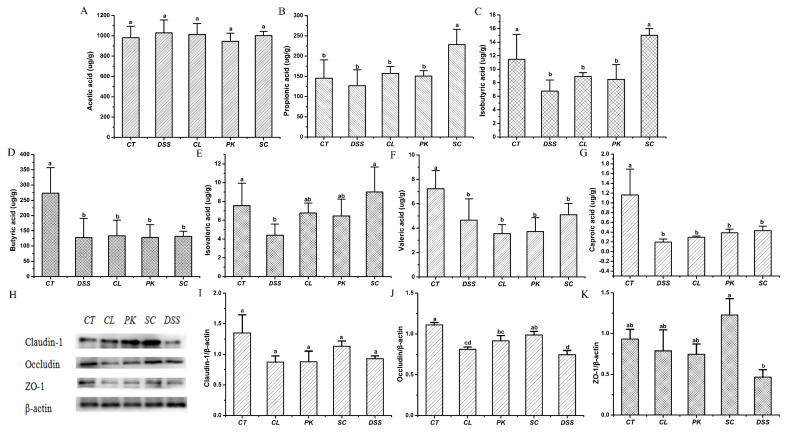
Effects of three yeast strains on the short-chain fatty acid contents and barrier proteins of the conlon in Balb/c mice in different groups. (**A**–**G**) Acetic acid, Propionic acid, Isobutyric acid, Butyric acid, Isovaleric acid, Valeric acid, Caproic acid; (**H**) Western blotting images of Claudin–1, Occludin and ZO–1. Relative expression of (**I**) Claudin 1, (**J**) Occludin, (**K**) ZO–1. Different lowercase letters marked on the column represent significant difference (*p* < 0.05).

H&E staining was used to evaluate the colon histopathological injury of the Balb/c mice with enteritis. As shown in Figure 2D-1, the normal Balb/c mice had regular crypt structure, complete mucosal layer structure, uniform distribution of goblet cells and neat arrangement of glands in the colon tissue. However, the crypt structure was destroyed or disappeared, goblet cells decreased significantly and a large number of inflammatory cell infiltration phenomena were found in the colon tissue of the mice in the model group (Figure 2D-2). These tissue injuries were well repaired after oral administration with the yeast of *S. cerevisiae* I4, showing a better ameliorating effect than the other two yeast strains (Figure 2D3-5). A large number of clinical studies have confirmed that inflammatory cell infiltration and intestinal barrier damage are the key factors leading to colitis [39]. Impaired intestinal barrier function, significant reduction of mucins and tight junctions are important pathological features of colitis patients [40,41]. As shown in Figure 2E-1,E-2, compared with the blank group, goblet cells and mucins in the colon tissues of the mice in the model group were greatly reduced for there was almost no blue. The number of mucins dyed blue increased significantly after oral administration with the yeast of *P. kudriavzevii* 11 and *S. cerevisiae* I4, suggesting that yeasts could positively affect intestinal barrier function by increasing the content of mucins. It’s worth noting that *S. cerevisiae* I4 was the most effective in alleviating the symptoms of colitis (Figure 2E-5).

### 3.5. Effect of Yeasts Oral Administration on Inflammatory Mediators and Immune Related Factors of the Balb/c Mice with Enteritis

Thymus and spleen are important immune organs and organ index can reflect the body’s non–specific immune function to some certain extent [42]. As shown in Figure 3A–C, the thymus atrophy and spleen enlargement of the Balb/c mice with enteritis caused by DSS were ameliorated after oral administration of three yeasts, indicating that yeasts could regulate the immune function of the mice with colitis to a certain extent. Among them, *S. cerevisiae* I4 had attracted our attention because it performed the best role in improving thymus atrophy.

Four inflammatory factors and three immunoglobulin in serum of the Balb/c mice were determined by ELISA kit in order to detect the effects of the three yeast strains on inflammatory mediators (Figure 3D–G). The pro-inflammatory cytokines expression of the Balb/c mice in the model group after drinking DSS water, including TNF–α, IL–6 and IL–1β, increased significantly compared with those in the blank group (*p* < 0.05), but the contents of anti-inflammatory factors IL–10 and immunoglobulin declined (*p* < 0.05). After oral administration of the three yeast strains, especially the *S. cerevisiae* I4, TNF–α, IL–6 and IL–1β decreased to varying degrees (*p* < 0.05), while IL–10, IgM, IgA and IgG increased (*p* < 0.05). The results indicated that oral administration of yeasts alleviated colitis in the Balb/c mice by reducing pro–inflammatory cytokines and increasing anti–inflammatory factors and regulating immune status.

### 3.6. Regulation of Intestinal Microflora of the Mice with Colitis by Yeasts

Intestinal gut microbiota disorder is a key factor leading to inflammatory bowel disease (IBD), mainly by inducing abnormal immune response of intestinal mucosa [43]. The Shannon, Chao1 and ACE index are used to reflect the diversity and richness of communities. It can be seen from Figure 4A that the Shannon index of the model group was reduced compared with the control group (*p* < 0.05), indicating that the diversity of intestinal gut microbiota in mice was decreased. However, the value increased after intragastric administration of *S. cerevisiae* I4 although there was no significant difference. In addition, for the three yeast strains, *C. lusitaniae* 30 and *P. kudriavzevii* 11 can better alleviate the decline of microbial richness caused by colitis (Chao 1, *p* > 0.05; ACE, *p* < 0.05) (Figure 4B,C).

The differences of intestinal gut microbiota between groups were further demonstrated by principal coordinate analysis (PCoA) and non-metric multidimensional scaling (NMDS). As shown in Figure 4D, the DSS model group was distinctly far away from the other groups, and the microbial community composition structure was quite different from the normal group, which implied that the stability of intestinal gut microbiota decreased and the microbiota structure was destroyed after inducing colitis. In contrast, the three yeast groups were close to and crossed with the blank group, suggesting that the microbial community composition of mice after yeast treatment was close to the normal group.

The effects of yeast on the relative abundance of intestinal gut microbiota in mice with colitis were evaluated at different levels by high-throughput sequencing. At the phylum level, the proportion of Firmicutes increased and Bacteroidetes decreased in DSS model group compared with the control. Notably, the relative abundance of Proteobacteria and Actinobacteria uplifted although there were no significant differences. After intragastric administration of yeast, the proportion of Proteobacteria and Actinobacteria associated with colon inflammation was reduced by repairing the intestinal gut microbiota (*p* > 0.05) (Figure 4F). At the family level, the abundance of beneficial bacteria, such as Muribaculaceae (*p* > 0.05), Lactobacillaceae (*p* > 0.05) and Rikenellaceae (*p* < 0.05) diminished in the DSS model group. Nevertheless, the proportion of Lachnospiraceae that has been reported as a potential pathogen of DSS induced mice raised, the study by Mu et al. [44] also confirmed this result. The proportion of beneficial bacteria such as *Lactobacillus* (*p* > 0.05), Muribaculaceae (*p* > 0.05) and Rikenellaceae (*p* < 0.05) increased to varying degrees after the treatment of the three yeast strains, while the abundance of Lachnospiraceae decreased (*p* < 0.05) (Figure 4G). At the genus level, *Lactobacillus*, a common probiotics that can produce short-chain fatty acids, inhibit the growth of various pathogenic bacteria and maintain the balance of intestinal gut microbiota was reduced in the DSS model group (*p* > 0.05) [45]. The abundance of harmful bacteria such as *Staphylococcus* and *Turicibacter* increased (*p* > 0.05). Yeasts, especially *C. lusitaniae* 30, augmented the relative abundance of *Lactobacillus* (*p* > 0.05), while *S. cerevisiae* I4 increased the proportion of *Paraprevotella* (*p* > 0.05) and SCFAs–producing *Odoribacter* (*p* < 0.01). Meanwhile, *S. cerevisiae* I4 and *P. kudriavzevii* 11 reduced the proportion of *Staphylococcus* (*p* > 0.05). These results proved that yeasts could alleviate the destruction of intestinal gut microbiota in mice by increasing the proportion of beneficial bacteria and reducing the proportion of harmful bacteria.

### 3.7. Yeasts Increased the Contents of SCFAs in Feces of the Mice with Colitis

SCFAs play a positive role in regulating host health, such as providing energy for the host, protecting and regulating intestinal epithelial barrier function and reducing inflammation [46,47]. As shown in Figure 5A–G, the contents of SCFAs such as Isobutyric acid, Butyric acid and Caproic acid (*p* < 0.05) in the intestine of mice drinking DSS water was significantly lower than that of the control group, which was verified by the results of previous studies [48]. After intragastric administration of *S. cerevisiae* I4, the production such as Propionic acid, Isobutyric acid and Isovaleric acid (*p* < 0.01) increased markedly, while Butyric acid, Valeric acid and Caproic acid showed an increase but no significant difference. In contrast, the other two strains had less effect on boosting SCFAs content.

### 3.8. Effect of Yeasts on Intestinal Barrier Function in the Mice with Colitis

Tight junction proteins such as Claudin–1, Occludin and ZO–1 are important components of the intestinal barrier and key structures to maintain the homeostasis of epithelial cells. Increased intestinal permeability or impaired intestinal barrier function caused by decreased TJP contents will aggravate the development of colitis [49]. In order to explore the effect of yeasts on intestinal barrier function in mice with colitis, the relative expression levels of Claudin–1, Occludin and ZO–1 proteins were determined. Figure 5H–K showed that the protein contents of Claudin–1, Occludin and ZO–1 in mice after drinking DSS water was lower than that in the blank group (*p* > 0.05), while the expressions showed an upward trend after treatment by three yeast strains, but there was significant difference only after treatment of *S. cerevisiae* I4 (*p* < 0.05).

## 4. Discussion

Fungi make up a relatively small percentage of the microbes that live in the human gut, but a variety of fungi including yeast are also involved in the steady–state regulation of the gastrointestinal tract in humans and other mammals [50]. Yeast is traditionally involved in food fermentation processes, including traditional fermented dairy foods, bread and so on, which has a long history of safe consumption. In recent years, many yeast strains with probiotic function have been developed and applied widely. Amandine et al. found that the *Saccharomyces boulardii* intervention could regulate the gut microbiota, alleviate the fat mass and hepatic steatosis in obese and type 2 diabetic mice [51]. Xu et al. confirmed that yeast β-glucans could remarkably reshape the intestinal gut microbiota and produce beneficial SCFAs in Aβ_1–42_-induced Alzheimer’s disease (AD) mice [52].

In this study, three yeast strains, isolated from traditional fermented dairy food in Xinjiang, China, showed different probiotic functions. Firstly, they all displayed strong tolerance to the artificial simulated gastrointestinal juice (74.6–77.0% survival rate). Since the gastrointestinal fluid of human beings is not conducive to the survival of probiotics and even lead to their death, the tolerance of probiotics to gastrointestinal fluid is a prerequisite for the colonization of strains with probiotics potential [34,53,54]. Greppi et al. isolated one *Pichia kudriavzevii* strain from African fermented cere-al-based foods, which showed the tolerance to low pH, 0.3% of bile salts and simulated gastrointestinal digestion (survival rate was 45%) than other strains, and combined with *Lactobacillus fermentum* strains to improve the production of folic acid in pearl millet porridge with its probiotic features [25]. Furthermore, Cho et al. reported that four strains of *Kluyveromyces marxianus* yeasts from kefir showed 5–25% greater intestinal adhesiveness than that of *Lactobacillus acidophilus*, among which, KU140723–02 (KM2) exhibited the greatest antioxidant activity and may be a potential functional food ingredient with antioxidant properties targeting gut health [55].

In our study, Balb/c mice aged 6–8 weeks showed weight loss, stool thinning, bloody stool, dim hair, reduced food intake and activity as predicted after drinking water with 3% DSS, the typical symptoms of colitis. Colonic shortening and activated neutrophil infiltration are regarded as significant markers of colitis [56]. *S. cerevisiae* I4 alleviated the histopathological characteristics such as colon shortening and mucosal structure damage of the Balb/c mice. The abnormal immune response caused by the imbalance of immune regulation is considered to be a vital part of the pathogenesis of UC [57]. In this study, the contents of IgA, IgG and IgM in serum of the mice oral administrated with *S. cerevisiae* I4 were significantly higher than those in the model group. IL–10 is an important anti-inflammatory cytokine that inhibits the production of pro-inflammatory cytokines such as IL–6 and TNF–α [56]. In the present study, oral administration of *S. cerevisiae* I4 increased the serum level of IL–10 and decreased the serum level of pro-inflammatory cytokines IL–6, TNF–α and IL–1β of the Balb/c mice induced by DSS, indicating that the strain could inhibit the abnormal pathogenic immune response in the mice with colitis. It was reported that β–glucan derived from yeast cell wall showed an immune regulating effect, which is recognized as a potential immunomodulator to strengthen innate and adaptive immunity [52]. Qi et al. confirmed that yeast-derived β–glucan activated dendritic cells (DCs) and macrophages via a C–type lectin receptor dectin–1 pathway, which could trigger effective anti-tumor immune response and notably down regulate immunosuppressive cells, resulting in delayed tumor progression [58]. Han et al. assessed the effect of oral administration of *Saccharomyces cerevisiae* on DSS-induced colitis in mice and found that the anti-inflammatory properties of this yeast were related to the β–glucan in its cell wall, which is a polysaccharide with immunomodulatory effect that inhibited the overexpression of DSS-induced pro-inflammatory factors including TNF–a, IL–6 and IL–8 [39]. The β-glucan of *S. cerevisiae* I4 should be extracted and assessed for the immune regulating effect.

Intestinal gut microbiota imbalance has been proved to be fundamentally associated with inflammatory bowel disease [59]. In this study, the gut microbiota disorder was manifested in the decrease of the diversity and richness of intestinal flora in the Balb/c mice of the model group. The increased proportion of Proteobacteria (2.87–5.11%) and Actinobacteria (1.36–2.38%) in model mice was also an obvious feature, while oral administration of three yeasts can reduce the abundance of these two harmful bacteria, which are dominant in the colon of UC patients. For the family level, Muribaculaceae is one of the major intestinal gut microbiota identified in healthy individuals, which can produce Succinic acid, Acetic acid and Propionic acid [44]. *Lactobacillus* is recognized as a probiotic and can reduce DSS–induced colon inflammation and injury [60]. *S. cerevisiae* I4 treatment increased the proportion of *Lactobacillus*, Muribaculaceae and Rikenellaceae, which was consistent with the results of Liu et al. [61]. On the other aspect, some intestinal microorganisms can ferment dietary fiber to produce SCFAs, mainly including Acetic acid, Propionic acid and Butyric acid, which have been shown to regulate intestinal homeostasis and ameliorate inflammatory bowl disease [62,63]. Our study showed that the contents of SCFAs such as Acetic acid and Propionic acid of the feces in the mice treated with three yeast strains were significantly augmented, which was also related to the increase of beneficial bacteria such as Muribaculaceae, *Lactobacillus* and Rikenellaceae, because these bacteria were associated with fiber degradation and could affect the development of colitis by increasing the level of intestinal SCFAs. Coker et al. reported that SCFAs participate in the homeostasis of immune response and is an effective anti-inflammatory agent for inflammatory bowel disease [64].

Tight junction proteins and mucins are the main components of intestinal barrier, their reduction or loss is closely related to the occurrence and development of colitis [65,66]. Colonic mucosa is an important defense barrier, in which MUC2 is the main component of mucus layer [40,67]. In this study, Alcian blue staining showed that the goblet cells and secretion of MUC2 in the colon of the mice with colitis were substantially reduced compared with those in the control group. Van der Sluis M et al. reported that the MUC2 gene knockout mice showed stronger susceptibility to DSS induced colitis, due to the direct exposure of epithelial cells to pathogenic microorganisms [68]. While, oral administration of *S. cerevisiae* I4 enhanced the relative expression of intestinal barrier proteins Claudin–1, Occludin and ZO–1 of the Balb/c mice. Intestinal epithelial cells are connected by tight junction proteins, which play an indispensable part in controlling intestinal permeability and maintaining intestinal barrier function. Liao et al. found that both fructooligosaccharides (FOS) and synbiotic supplementation could alleviate DSS–induced acute colitis of the mice by reducing the inflammatory potential of the host and restoring the expression levels of MUC2 and TJPs including Occludin, Claudin–1 and ZO–1 [56]. Obviously, the increase of intestinal permeability accelerates the passage of harmful pathogens when the tight junction structure is destroyed, thereby aggravating the development of inflammation. In addition, SCFAs such as Propionic acid and Butyric acid can strengthen the intestinal barrier, inhibit inflammation and inflammatory signal transduction of immune cells as the main energy source of intestinal epithelial cells [69,70,71].

## 5. Conclusions

*S. cerevisiae* I4, isolated from traditional fermented dairy food in Xinjiang, showed good gastrointestinal fluid tolerance and adhesion to HT–29 cell monolayers without any toxicity. Furthermore, oral administration of *S. cerevisiae* I4 ameliorated the symptoms such as diarrhea, colon shortening, and histological damage including inflammatory cell infiltration in the Balb/c mice with colitis, which are attributed to that *S. cerevisiae* I4 regulated intestinal gut microbiota balance and upgraded intestinal barrier function by increasing the expression of TJPs and mucins. In general, *S. cerevisiae* I4 showed promising potential as probiotis to alleviate symptoms of UC, which provides a new direction to explore yeast resources in the traditional fermented dairy products from Xinjiang to obtain beneficial yeast applied in functional food.

## Figures and Tables

**Table 1:** Survival rate of three yeast strains in simulated gastrointestinal juice and adhesion rate to HT–29.

Strains	Survival Rate (%)		Adhesion Rate
	Gastric Juice	Intestinal Juice	
*Clavispora lusitaniae* 30	86.4 ± 0.020 ^a^	77.0 ± 0.018 ^a^	93.2 ± 0.006 ^b^
*Pichia kudriavzevii* 11	88.0 ± 0.006 ^a^	74.6 ± 0.013 ^a^	96.8 ± 0.008 ^a^
*Saccharomyces cerevisiae* I4	88.4 ± 0.013 ^a^	76.7 ± 0.016 ^a^	96.0 ± 0.011 ^a^

Different letters represent significant differences (*p* < 0.05)

**Table 2:** Effects of three yeast strains on HT–29 cell viability.

Concentrations	Cell Viability(%)		
	*Clavispora* *Lusitaniae* 30	*Pichia* *kudriavzevii* 11	*Saccharomyces cerevisiae* I4
10^5^	91.45 ± 3.05 ^b^	116.29 ± 2.47 ^b^	100.8 ± 2.36 ^b^
10^6^	96.92 ± 2.07 ^ab^	121.60 ± 3.34 ^b^	117.15 ± 5.05 ^a^
10^7^	101.62 ± 5.74 ^a^	133.08 ± 7.85 ^a^	126.66 ± 9.52 ^a^

Different letters represent significant differences (*p* < 0.05)

## Data Availability

Data is contained within the article.
